# Penetrating Gunshot Wound of the Abdomen

**Published:** 1887-08

**Authors:** Edmund Andrews

**Affiliations:** Professor of Clinical Surgery, Chicago Medical College


// THE MEDICAL JOURNAL AND EXAMINER.

VOL. LV. AUGUST, 1887. NO. 2.

### PENETRATING GUNSHOT WOUND OF THE ABDOMEN, WITH A REPORT OF FOUR CASES.

BY EDMUND ANDREWS, M. D., LL.D., PROFESSOR OF CLINICAL SURGERY, CHICAGO MEDICAL COLLEGE.

CASE I.—A lady sitting in her house was struck by a stray bullet coming in at the window, on the Fourth of July last. The bullet entered about two inches downward and outward from the anterior superior spinous process of the ilium. A thoroughly skillful family physician examined the wound and found appearances pointing to the probability that the bullet had traversed obliquely downward in a direction where it could not injure the viscera, so that strong hopes were entertained that nothing dangerous would result. On the following morning, however, he discovered decided evidences of peritonitis, and sent for me in consultation. We carefully examined the patient and agreed on the necessity of an exploratory operation, which was commenced about sixteen hours after the injury. The patient, aside from the peritonitis, seemed in a fairly good condition for enduring operative procedures.

Such antiseptic measures and precautions as could be taken without too much loss of time, were rapidly resorted to. The operating room was cleared out and scrubbed with antiseptic solutions. The patient's abdomen was washed thoroughly with a similar solution, likewise the hands, hair and faces of the operator and assistants, and clean laundried linen drawn on over the other clothing, wet with antiseptics, etc. A small incision was made in the linea alba below the umbilicus, which showed that the abdomen was full of bloody serum. The opening was moderately enlarged, and the serum removed with great care, when the intestines on the side towards the wound were seen to be intensely red with inflammation, and partially agglutinated with fresh adhesions.

No particles of any material could be found among the intestines, or floating in the serum giving any appearance of having escaped from the alimentary canal. The small intestine was carefully examined by passing it through the finger, and at a point a little above Poupart's ligament, on the left side, an opening was found in it, and also an orifice through the mesentery of an adjacent coil. The wound was closed with Lembert's suture, using antiseptic silk for the stitches.

At this point the patient's pulse began to fail. Search, however, was continued until it was reasonably certain that no other opening in the intestine existed. We then began a complete "toilet of the peritoneum" by clearing out all the remaining bloody serum. No clots of any considerable size were found, and the actual quantity of blood effused was not large. Before this process could be finished to my entire satisfaction, the pulse of the patient sunk still lower and became almost imperceptible, so that she seemed likely to die on the table from shock. The cleansing of the abdominal cavity was therefore finished tolerably well, but not with the thoroughness that would otherwise have been done. The wound was dressed antiseptically, in the usual manner, and the patient placed in bed and treated with warmth and stimulants. After some hours she reacted. The next morning all the symptoms were more favorable. The temperature was lower than before the operation and the strength seemed comparatively good. The following day, which was the third from the injury, she sunk into a state of asthenia and died. A proper autopsy was not obtained, but a slight examination of the abdominal cavity showed some degree of renewal of the serous effusion without any material hæmorrhage.

CASE II. was another fourth of July accident.

A robust man of middle age, being engaged in a dispute with another person, the latter drew a pistol and shot him in the left side of the abdomen, a little above the superior spinous process of the ilium, penetrating the cavity of the abdomen.

Great shock followed, and, on evacuating the urine, it was found to be bloody, indicating that the left kidney had been struck by the bullet. Sixteen hours afterwards I was called in consultation. The patient was moribund, the pulse almost imperceptible, and at moments when it could be counted it was about 150. Deeming that he was already past any possibility of help, no laparotomy was attempted.

CASE III.—A young man going past a saloon, whilst a row was in progress, but on the op[illegible]site side of the street, received a shot from a small pistol fired by some one in front of the saloon. Being called in consultation, on the same day, I found the pistol-wound directly over the stomach in the linea alba, above the umbilicus. As this was many years ago, laparotomy was not considered among the recognized remedies for the condition. The patient was kept in bed, given small quantities of water at a time for drink and deprived for some days of food. He was also kept pretty thoroughly under the influence of opiates. No bad symptoms of any kind occurred. About the tenth day he passed the bullet per anum, and recovered as if his injury had been of the most trivial sort.

CASE IV.—A robust young man was in a saloon where a policeman attempted an arrest, and in doing so used his pistol, having a calibre of 42. The bullet struck the man in his back, a little above the right kidney, and passed out at the linea alba, halfway between the umbilicus and the extremity of the xiphoid cartilage.

The young man's stomach was empty of food, but he had taken a pint or two of beer. He vomited blood and beer. Considering the direction of the bullet and the vomiting of the blood, I deemed it reasonably certain that the stomach was perforated and recommended laparotomy to him. This he promptly refused to submit to, so I confined him to bed, deprived him of food, and gave him opiates in order that he might die a correct and scientific death. However, nothing particular occurred. No fever, inflammation or peritonitis followed, nor anything else of any consequence whatever. The patient recovered with more ease and comfort than he would have done from the amputation of a finger.

I am not prepared to make an elaborate discussion upon these cases, for evidently what we need at present is an accumulation of facts upon which to base future reasonings. The important question which presses for answer in such cases is this: Shall there be a laparotomy? My opinion is that where an intestine is wounded a laparotomy is usually demanded, if the patient is not already in too prostrate a condition to give hope of success.

But the more difficult class of cases are those apparently in which the stomach is wounded with small bullets.

The author of the "Surgical History of the War of the Rebellion" expresses the opinion that wounds of the stomach not laparotomized will be found more dangerous than those of the intestines.

My own observation would tend in a contrary direction. It is my opinion that the thick walls of the stomach, lined with a tough mucous membrane loosely attached to it, admits of being perforated in many instances by a small bullet without leakage of the contents. The muscular fibers of the thick walls of the stomach seem to spread and close together again after the bullet, as they would after an aspirator needle. At the same time, where the shot is oblique the bullet may traverse a little distance between the muscular walls and the mucous lining, so that the openings of the two are not opposite each other, making a valvular arrangement which prevents leakage. On the contrary, the thin walls of the intestine do not admit of such a result, and leakage is inevitable if they are perforated.

Unfortunately no man can distinguish positively between the conditions beforehand; and though the stomach wounds often recover without operation, it is best to make at least an exploratory opening into the abdomen in most cases of penetrating wounds, unless the general condition is such as renders it morally certain that the patient cannot survive the shock.

In military practice, and before abdominal section was used for gunshot wounds, the mortality from this injury was about 76 per centum, exclusive of those who died at the line of battle, without appearing at the field depot. The small pistol shots of civil practice are probably less fatal than the ounce-weight military projectiles, so that apparently the average patient has more than one chance in four of recovery without operation; yet if we could select out the intestinal gunshot wounds and reckon their results separately, we should probably not find one in twenty recovering, and these are the ones in which laparotomy is the most urgent. However, no one can determine beforehand precisely what the bullet has cut, hence, as before stated, the comparatively safe exploratory incision to determine the exact injury should be employed far more frequently than it now is.

